# Difficult removal: a Swan-Ganz catheter coiled on the central venous catheter

**DOI:** 10.1186/s13019-020-01149-4

**Published:** 2020-05-19

**Authors:** Jian Shen, Luyao Ma, He Huang

**Affiliations:** 1 Department of Anesthesiology, First Affiliated Hospital with Nanjing Medical University, Guangzhou Road 300, Nanjing, Jiangsu Province 210029 PR China; 2 Department of Cardiovascular Surgery, First Affiliated Hospital with Nanjing Medical University, Nanjing, China

**Keywords:** Swan-Ganz catheter, Complication, Difficult removal

## Abstract

**Background:**

The Swan-Ganz catheter plays an important role in gaining understanding of cardiac pathophysiology and in the hemodynamic monitoring of critically ill patients. Difficult removal of the Swan-Ganz catheter is a rare but serious complication.

**Case presentation:**

This case presents the difficult removal of a Swan-Ganz catheter in a 28-year-old female patient after cardiac surgery. Fluoroscopy and chest X-ray revealed that a portion of the Swan-Ganz catheter was coiled on the central venous catheter at the level of the superior vena cava. Under X-ray guidance, the central venous catheter was first removed, and then the Swan-Ganz catheter was successfully withdrawn through the percutaneous introducer sheath.

**Conclusions:**

This case report provides an unreported reason for difficult removal and describes a successful solution. This report suggests that X-ray examinations may be necessary before removing the Swan-Ganz catheter.

## Background

Difficult removal of the Swan-Ganz catheter has been reported with an estimated incidence of 0.2–2.5% [1], due to reasons including intracardiac knots and kinks in the Swan-Ganz catheter [2–6]. Cardiac perforation and pulmonary artery rupture were the most severe complications due to the difficult removal of a catheter in a previous report [7, 8]. This case report features the difficult removal of a Swan-Ganz catheter, which coiled on the central venous catheter in a patient after cardiac surgery.

## Case presentation

A 28-year-old woman, weight 55 kg and height 162 cm, initially presented to the emergency department of an outside hospital with a 2-month history of chest distress, cough and dyspnea. The patient’s symptoms did not improve significantly after oxygen therapy or anti-asthma medication combined with aerosol inhalation. The patient was later transferred to the cardiovascular surgery department in the authors’ institution with aggravation of the above symptoms. Echocardiography revealed severe tricuspid regurgitation and congenital heart disease, including a ventricular septal defect and rupture of the right coronary sinus aneurysm. An abdominal computed tomography scan revealed that the inferior vena cava and hepatic veins were widened. The preoperative laboratory examination was unremarkable. For invasive hemodynamic measurement, a Swan-Ganz catheter (Edwards Lifesciences, Irvine, CA) was inserted through an 8-F percutaneous introducer sheath placed in the right internal jugular vein, and a central venous catheter was inserted into the right subclavian vein without any difficulties. The pulmonary artery pressure waveform suggested that the catheter was successfully floated into the pulmonary artery. The patient underwent ventricular septal defect repair, aortoplasty and tricuspid valvuloplasty under cardiopulmonary bypass.

**Fig. 1:**
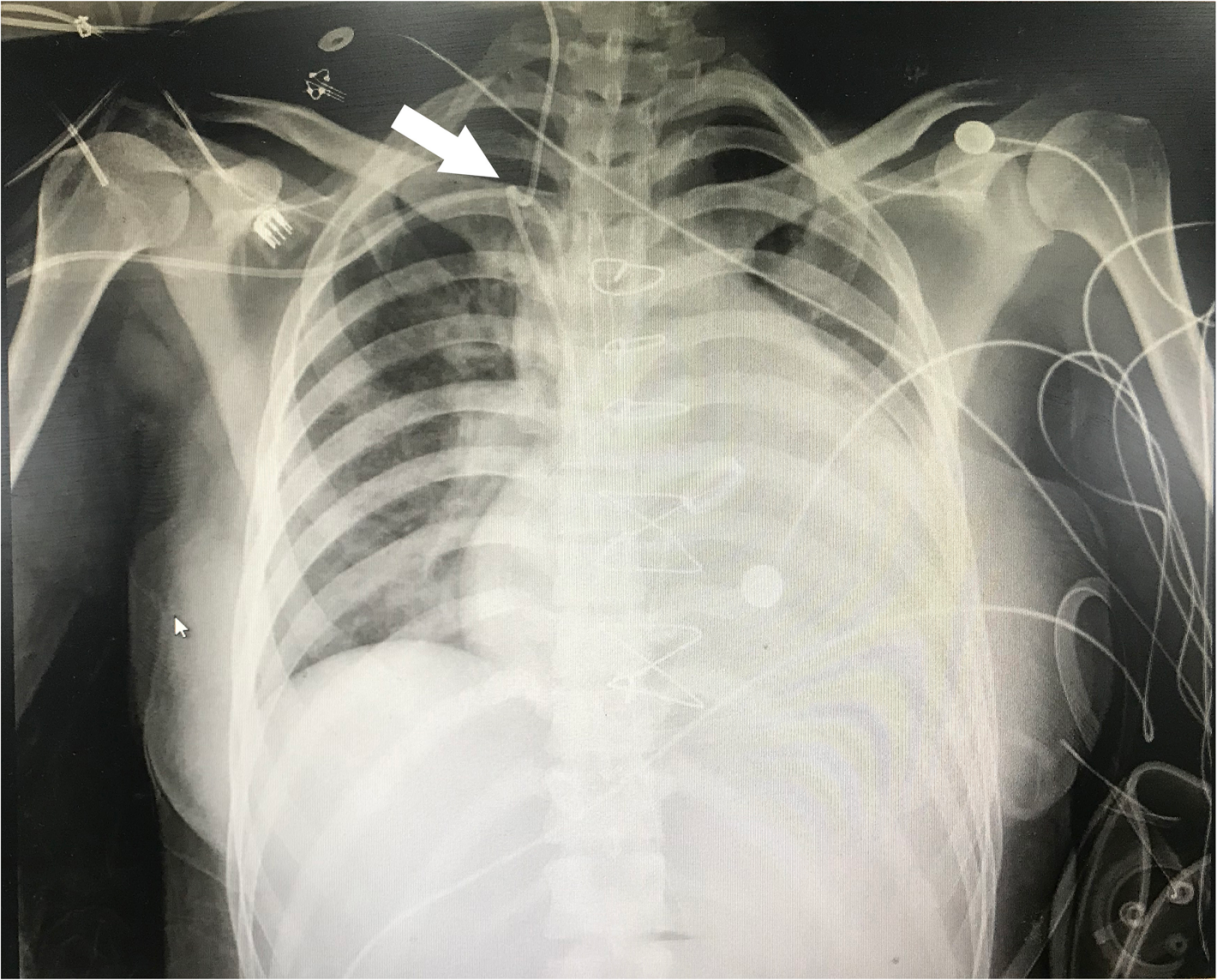
The Swan-Ganz catheter coiled on the central venous catheter at the level of the superior vena cava

**Fig. 2:**
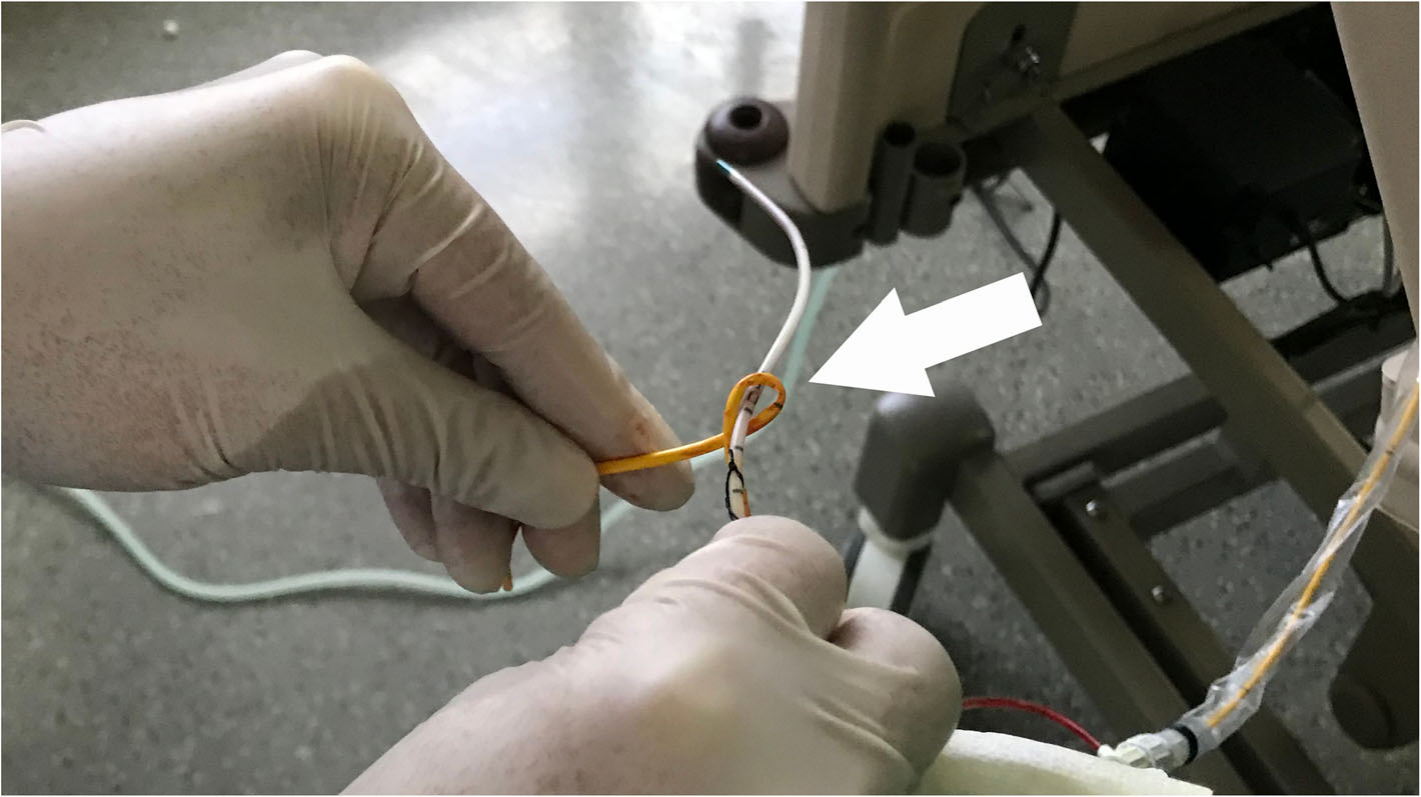
A closer inspection of the Swan-Ganz catheter and central venous catheter after removal

After obtaining the hemodynamic indexes postoperatively, the Swan-Ganz catheter was found difficult to withdraw when it was pulled out to 35 cm mark. The cardiovascular surgeons and intensive care physicians suggested transporting the patient to interventional radiology. The chest X-ray showed that a portion of the Swan-Ganz catheter coiled on the central venous catheters at the level of the superior vena cava and formed a knot approximately 1 cm in diameter (Fig. 1). Under X-ray guidance, the interventional radiologist first removed the central venous catheter smoothly. Then, the Swan-Ganz catheter was gently pushed into the right ventricle to provide more space to uncoil, and then the catheter returned to its original coiled configuration. Finally, the Swan-Ganz catheter was successfully withdrawn through the percutaneous introducer sheath. A closer inspection of the Swan-Ganz catheter coiled on the central venous catheter is shown in Fig. 2. During this procedure, the patient’s hemodynamics were not significantly affected, and the patient did not present any complications or cardiovascular injury. Valvar damage was excluded by echocardiography.

## Discussion and conclusion

The use of a Swan-Ganz catheter with balloon flotation is a feasible and rapid technique for hemodynamic monitoring in critically ill patients [9]. However, there have been continuous reports of complications related to the removal of Swan-Ganz catheter in clinical practice, and the common causes include intracardiac knots and kinks in the catheter (Table 1). In this case report, fluoroscopy and chest X-ray revealed that a portion of the catheter coiled on the central venous catheter and formed a knot. The cause of the knot is unknown, and the authors hypothesize that the catheter end may form a knot when the cardiothoracic surgeon reinserted the Swan-Ganz catheter into the distal portion of the pulmonary artery after performing tricuspid valve surgery under cardiopulmonary bypass. Therefore, the loop of the catheter may easily coil around the central venous catheter when the Swan-Ganz catheter is withdrawn. This case suggested that although pulmonary artery pressure wave may reflect the Swan-Ganz catheter successfully floated into the pulmonary artery, it could not indicate whether the catheter formed the intracardiac knot and kink.

**Table 1:** Difficult removal of the Swan-Ganz catheter

Cause	Treatment scheme	Citation
Knot	Retrieval of a knotted Swan-Ganz catheter using an interventional approach.	Shang et al., 2016 [2]
	Removal of the knotted Swan-Ganz catheter by using transesophageal echocardiography.	Yuan et al., 2010 [3]
	Attempts to open the knot percutaneously using a radiological approach failed; the knotted catheter was extracted with surgery.	Chemchik H et al., 2013 [4]
Kink	The catheter was pushed into the right ventricle, providing more room for the catheter to unfurl and allowing for the removal of the Swan-Ganz catheter.	Manoj et al., 2018 [5]
	Under direct fluoroscopic guidance, the interventional radiologist uncoiled the catheter by applying slow sustained traction on both the Cordis and Swan-Ganz catheters. Once the coil of the Swan-Ganz catheter approached the end of the Cordis catheter, both catheters were removed successfully simultaneously causing the coil to unwrap.	James et al., 2012 [6]

In this case report, the authors describe a successful management approach for removing a Swan-Ganz catheter. The key point for addressing loops and knots is to withdraw the central venous catheter with the assistance of radiologic techniques. Then, the Swan-Ganz catheter could be advanced into the right ventricle to provide more space for the catheter to uncoil. Finally, the catheter was withdrawn under X-ray guidance. This case report suggested that the surgeons should place the catheter in a normal coiled state before re-advancing the Swan-Ganz catheter intraoperatively to avoid forming knots and then gently place the catheter in the right ventricle. Additionally, it is important to take a chest X-ray before removing the Swan-Ganz catheter in patients undergoing tricuspid valve surgery. This process may help similar difficult removals of Swan-Ganz catheters, thereby eliminating the need for invasive maneuvers, but cannot be applied for tight knots.

## Data Availability

Not applicable.
